# Hospital physicians perform five types of work duties in Japan: An observational study

**DOI:** 10.1186/1472-6963-14-375

**Published:** 2014-09-06

**Authors:** Michiko Nohara, Toru Yoshikawa, Norihiro Nakajima, Kosuke Okutsu

**Affiliations:** Department of Hygiene and Public Health 1, School of Medicine, Tokyo Women’s Medical University, Tokyo, Japan; The Institute for Science of Labour, Kawasaki, Japan; Department of Hospital and Healthcare Administration, School of Medicine, Tokyo Women’s Medical University, Tokyo, Japan

**Keywords:** Work duties, Observational study, Physicians, University hospital

## Abstract

**Background:**

Physicians are expected to perform three unique roles as a clinician, educator, and researcher in university hospitals. However, the actual practices of physicians performing different duties are relatively unknown. Therefore, the authors conducted an observational study at a university hospital to examine physicians’ work activities.

**Methods:**

Between 2011 and 2013, ten observers shadowed 20 physicians from different specialties for a day at the Tokyo Women’s Medical University Hospital. Observers recorded physicians’ activities every 30 seconds that were subsequently categorized into work types. The number of work types and activity changes performed by a physician in one observational period were counted.

**Results:**

Authors categorized physicians’ work activities into five groups: patient care (direct and indirect), education, research, professional development, and administration. All physicians performed at least one type of activity in addition to patient care. Activity change occurred 1.86 times per hour, on average. The median time-distribution of 20 physicians was 173.8 minutes, 213.8 minutes, 3.3 minutes, 5.0 minutes, 0 minutes, and 0.8 minutes for direct patient care, indirect patient care, education, research, professional development, and administration, respectively.

**Conclusion:**

Japanese hospital physicians performed multiple work duties including professional development and administrative activities in addition to triple duties.

**Electronic supplementary material:**

The online version of this article (doi:10.1186/1472-6963-14-375) contains supplementary material, which is available to authorized users.

## Background

The term “triple role” or “triple duties” describes the characteristics of a physician’s work as a clinician, educator, and researcher in academic medicine [1–3]. Preparing for a lecture and obtaining grants for research, in addition to daily patient care is difficult and stressful [1, 3, 4]. This is also true for Japanese hospital physicians. In Japan, hospital physicians have been struggling with long working hours and heavy workloads [5]. Of 3,279 hospital physicians’ responses about their perception of workload, 68% (2,219) reported an increase in workload compared to the previous three years (Ministry of Health, Labour and Welfare, 2006) [6]. Duties other than patient care (62.2%), education/instruction (49.4%), and a growing number of outpatients (48.3%) and inpatients (32.7%) were found to be responsible for this increase. Prior research revealed a prevalence of long working hours among Japanese hospital physicians [7–9]. However, little has been reported on multiple duties of hospital physicians. Understanding the characteristics of hospital physicians’ work behaviors and the time they spend on them is the first step towards identifying areas for improvement and providing the necessary support.

Multiple aspects of physicians’ work often overlap with each other, and it may be difficult for physicians to compartmentalize these on busy working days. Time-motion studies, wherein a researcher directly observes and records the subject’s activity, are useful to objectively understand physicians’ work activities. Several time-motion studies in clinical settings have provided valuable data concerning physicians’ work in other countries [10]. In Japan, one such study investigated first- and second-year residents’ time allocation, thus the collected data did not necessarily cover the wide range of the physicians’ work activities [11].

In this study, we observed physicians with different specializations and experience, and categorized their work activities to demonstrate hospital physicians’ multiple work duties by direct observation.

## Methods

We conducted the observation study at a 1,423-bed university hospital in Tokyo, treating 1,181 inpatients and 4,185 outpatients, on average. Twenty physicians (10 females and 10 males, mean age = 36.3) were recruited from 20 departments for this study. In order to observe physicians who were responsible for clinical, educational, and research duties, inclusion criteria required that physicians worked full-time with over three years of experience. Upon receiving written consent, we observed each physician for a single day on randomly selected dates between October 2011 and January 2013.

Observers comprised four occupational physicians, five public health nurses, and one expert in ergonomics, who are qualified to correctly identify physicians’ activities. Three observers had prior experience conducting observational studies in clinical settings.

All observations took place on weekdays during the day, beginning at the physician’s arrival at the hospital, and ending at the physician’s end of duty. During the assessment, the observer wearing a white coat stood unobtrusively behind the physician (about 2 m) to avoid the Hawthorne Effect (where participants would be likely to change their behaviors due to the attention received from observers). The observer followed the physician to all locations, except when privacy was requested by either the physician or patient. The observer holding a clipboard and a watch shadowed each physician and recorded his or her main activity in 30-second increments on a specified paper form, along with the location and the companions [see Additional file 1.] Two or three observers alternated shadowing every two to three hours. We made sure to pair a novice with an experienced observer until the task was familiarized.

Generally for time-motion studies, an observer codes activities performed by a participant according to categories prepared beforehand based on a pilot study, to ensure the inter-observer reliability and the category’s comprehensiveness. In this study, however, we described physicians’ activities in a short sentence first, and categorized their activities after observation based on previous studies [11–14].

In order to examine physicians’ multiple work duties and work fragmentation, we counted the number of work types performed and activity changes during one observational period. Using the definition by Deshpande et al., we recorded activity changes only when a physician changed activities across categories [11]. We calculated each physician’s time distribution by category using Excel 2010 spreadsheets (Microsoft Cooperation^®^).

This study was approved by the Ethics Committee of Tokyo Women’s Medical University.

## Results

Demographic characteristics of the physicians are indicated in Table 1. We observed physicians for a total of 218 h, 44 min, and 30 s, with an average observation time of 10 h, 56 min, and 12 s per physician.

**Table 1:** **Demographic characteristics of physicians (n = 20)**

Characteristic					
Gender (n)	Female	10			
	Male	10			
Average age (years)	36.3				
Min–max (years)	27–55				
Position	Resident, Assistant professor, Associate professor				
Specialization	General Medicine		Pulmonology	Gastroenterology	Neurology
	Metabology		Endocrinology	Rheumatology	Radiation Oncology
	Pediatrics		Perinatology	Psychiatry	Emergency Medicine
	Cardiac Surgery		Plastic Surgery	Orthopedics	Obstetrics and Gynecology
	Urology		Otorhinolaryngology	Ophthalmology	Anesthesiology
Average observation time, h:min:s	10:56:12				
Min–max	4:51:00–14:06:00				

We categorized physicians’ work activities in five groups: patient care (direct and indirect), education, research, professional development, and administration.

1a) Direct patient care: Face-to-face interaction with patients; including activities such as medical consultation, history taking, treatment, ward rounds, and surgery.

1b) Indirect patient care: Behaviors integral to the treatment or management of patients performed in their absence; including medical conferences, case discussion with colleagues, charting, writing discharge summaries and insurance documents, and meeting patient’s family.

2) Education: Teaching activities, performed for both, medical school students and residents; tutoring, attending educational meetings, and short instruction delivery for residents were included.
3) Research: Activities related to clinical and basic research; including writing research papers, laboratory work, preparing manuscripts for medical meetings, and meeting for clinical trials.
4) Professional development: Activities for reinforcing knowledge and improving techniques related to one’s specialty, or to general clinical care; activities such as attending lectures, receiving instructions from senior physicians, watching senior physicians’ outpatient service, small group studies, reading articles, attending conferences with medical representatives, and attending medical meeting rehearsals were included.
5) Administration: Obligatory non-medical work for both departmental and hospital operation; including administrative conferences, checking mails, and completing administrative paperwork.

The number of work types that physicians performed in one observational period is described in Table 2. Half of the physicians performed more than four different types of work in one day and changed activities across categories 1.86 ± 0.95 times per hour on average (0.7–4.5) in one observational period. Activity change included changes between direct and indirect patient care, excepting the repeated changes occurring between outpatient visits.

**Figure 1:**
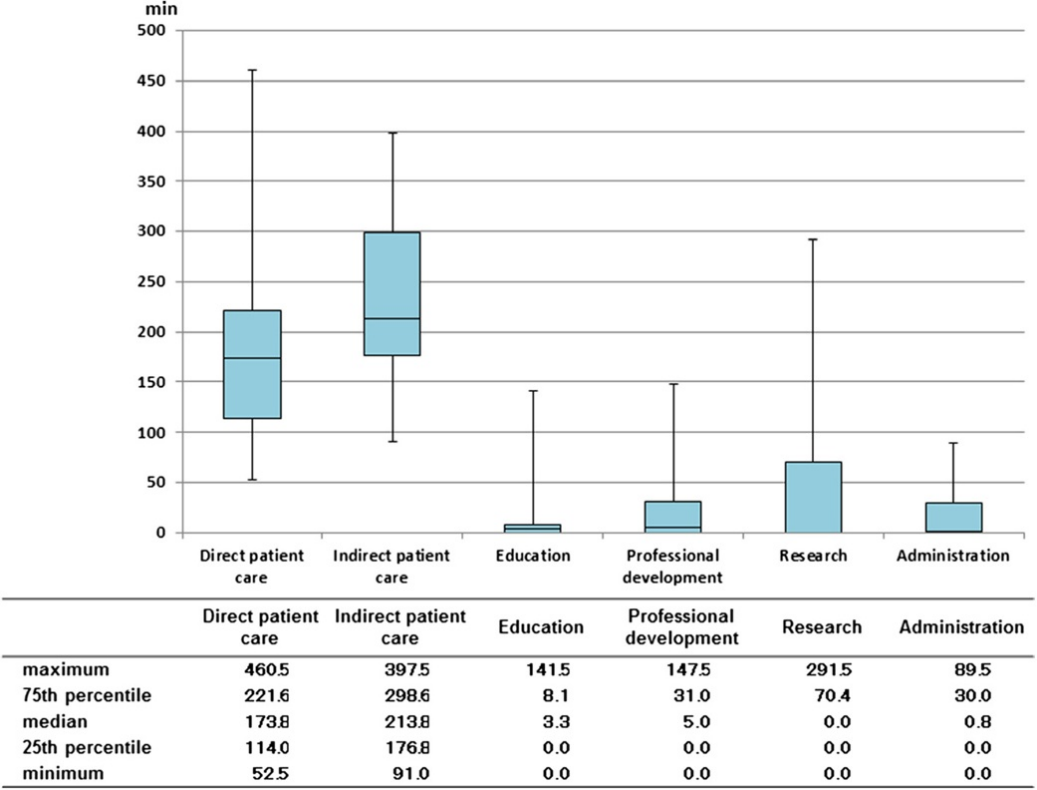
**Distribution of physicians’ time spent on each work type.** Box plots denoting time-distribution of 20 physicians’ for patient care, education, research, professional development and administration.

The distribution of time for each category (work type) is shown in Figure 1.

**Table 2:** **Number of work types performed by 20 physicians at one observation**

	Patient care direct/indirect	Education	Research	Professional development	Administration	Total	Observation time (min)
1	✓	✓		✓	✓	4	707
2	✓	✓	✓	✓		4	846*
3	✓		✓	✓	✓	4	737.5
4	✓	✓				2	618.5
5	✓		✓	✓		3	638*
6	✓	✓	✓	✓		4	592
7	✓	✓				2	620
8	✓	✓	✓		✓	4	743*
9	✓	✓	✓			3	291
10	✓	✓			✓	3	666.5
11	✓			✓		2	618.5
12	✓	✓		✓		3	631
13	✓		✓	✓	✓	4	813
14	✓	✓	✓		✓	4	576.5*
15	✓	✓				2	540.5*
16	✓	✓		✓	✓	4	734*
17	✓			✓	✓	3	630
18	✓			✓		2	737
19	✓	✓		✓	✓	4	614
20	✓	✓	✓	✓	✓	5	770.5

Note: *indicates the physicians who spent additional time at the hospital before or after the observation.

## Discussion

This study objectively delineates the multiple duties of Japanese hospital physicians by categorizing physicians’ work activities into five groups. As expected, physicians performed work tasks commensurate with “triple duties”: patient care, education, and research. This study demonstrated that physicians performed two additional tasks: professional development and administration. Since physicians are expected to continually keep abreast with current research, professional development may be considered a personal goal. However, some activities observed in this study like attending conferences with medical representatives and medical meeting rehearsals, were scheduled into physicians’ routines for which they were obliged to participate. Therefore, we regarded it appropriate to categorize professional development as a duty.

All physicians regularly performed at least one additional type of activity, whether education, research, professional development, or administration, in addition to patient care within one observational period, indicating that they performed multiple work duties. The average activity change across categories was 1.86 times per hour. This was less frequent than the previous study on first- and second-year residents that obtained an average of 2.83 times per hour [11]. This may be a result of excluding the changes between direct and indirect patient care during outpatient services, and the greater control the physicians in our study had over their work as compared to the residents in the previous study. Patient care, taking more than half of physicians’ time, was the primary task among the others. The growing demands for advanced, safe care and the pressure of clinical administrative work have made physicians’ clinical load heavier [15], depriving physicians time for other duties. Therefore, hospital administrators should attempt to reduce physicians’ clinical workload, taking into account their multiple work duties.

The physicians’ time allocation varied widely on the basis of specialization, consistent with research by Gabow et al. [13], who reported differing work activities and time allocation for residents based on the residents’ four specializations. As some previous researches have shown [10], this study also has shown that the longer time dedicated to indirect patient care than to direct patient care.

In the present study, instead of immediately coding the activities performed by physicians, we recorded these in detail. While the descriptions were time consuming, they allowed us to carefully review each work activity to make precise categorizations. Some activities in particular required careful categorization; for example, grand rounds, unlike daily rounds, have greater implications for education rather than patient care. Therefore, even if grand rounds were performed in the presence of the patient, they should be categorized under professional development or education depending on the position of the physician. Additionally, it was difficult to categorize charting that was not conducted in a consultation room, as it could have been categorized under indirect patient care, professional development, or research. To make this distinction, physicians were asked about the nature of the activity when it was undistinguishable, immediately after observation. Observers described activities in detail with an acute understanding of hospital physicians’ work; this enabled us to categorize activities taking into account the physician’s position and situation, and to present time allocation in ways that reflected various implications of work activities depending on physicians.

Through the observation of 20 physicians, we found that six physicians spent additional time in hospital before or after their duty hours (Table 2). We started and ended the observation according to each physician’s instruction. However, one physician arrived at hospital before the appointed time, and five physicians did not leave hospital soon after the observation ended. They spent an average of 92.4 minutes (40–160.5) at the hospital before or after the observation. They regarded the time as personal time and distinguished it from duty hours. However, one of the physicians answered that he was reviewing the outpatients’ data of that day, which could have been recorded as indirect patient care. This may imply the voluntary overtime work of hospital physicians [1]. Similarly, working at home was reported in other study [16], suggesting future studies are needed to evaluate such physicians’ voluntary work more properly.

While the study provides useful findings, there are 4 limitations. One limitation is that we have not tested the interrater reliability prior to the main study although we guaranteed observers’ ability to identify physician’s activities. Another limitation is that the problems of generalizability of results since physicians from a single university hospital were observed for a single day. However, our data reflected the hospital physicians’ work as a whole and its various aspects since we selected the observation days at random and selected physicians with different specializations. Further, work activities performed by a physician at different points during their career were considered by observing physicians with differing experience levels. While it took one year and three months to complete all 20 observations to match physicians’ and observers’ schedules, the observation conditions remained consistent since no institutional changes were made. The other limitation was the exclusion of the time physicians spent during night duty, an essential part of hospital physicians’ work time. An observational study including a night duty component could lead to greater insights.

## Conclusion

This study revealed that Japanese hospital physicians performed five different types of work. In addition to the triple duties of physicians (patient care, education and research), they also devoted their time to professional development and administrative work. Our findings have provided an objective information for the better understanding of physicians’ heavy workload which has been mainly discussed as personal impressions in the literature. Hospital administrators should know the various duties for which hospital physicians are responsible when the administrators attempt to reduce physicians’ workload.

## Electronic supplementary material

Additional file 1: **Data Sheet.** (XLSX 13 KB)
